# Nucleophilic Substitution on 2-Monosubstituted Quinoxalines Giving 2,3-Disubstituted Quinoxalines: Investigating the Effect of the 2-Substituent

**DOI:** 10.3390/molecules21101304

**Published:** 2016-09-30

**Authors:** Ndumiso Thamsanqa Ndlovu, Winston Nxumalo

**Affiliations:** Department of Chemistry, Faculty of Science and Agriculture, University of Limpopo, Private Bag X 1106, Sovenga 0727, South Africa; nthami001@gmail.com

**Keywords:** 2,3-disubsituted quinoxaline, nucleophilic substitution, oxidative rearomatization

## Abstract

An investigation on the effect of substituent at the 2-position of mono-substituted quinoxalines in the synthesis of di-substituted quinoxaline derivatives via nucleophilic substitution reactions, is reported. Di-substituted quinoxalines bearing aryl-alky, aryl-aryl, aryl-heteroaryl, aryl-alkynyl, and amino-alkyl substituents were prepared in moderate to good yields. 2-Monosubstituted quinoxalines bearing a phenyl and butyl substituent reacted readily with alkyl-, aryl-, heteroaryl- and alkynyl- nucluephiles, giving di-substituted quinoxalines. 2-Monosubstituted quinoxalines bearing an amine and alkynyl substituent only reacted with alkyl nucleophiles. Oxidative rearomatization to give 2,3-disubstituted quinoxaline products occurred in atmospheric O_2_.

## 1. Introduction

Quinoxaline derivatives possess extensive applications in medicinal chemistry, due to their broad spectrum of biological activity [1,2]. A large number of synthetic quinoxalines have been reported to exhibit anti-tubercular [3], anti-viral [4,5], anti-microbial [6,7], and neuroprotective [8,9] activity. Quinoxaline derivatives have been reported to be prepared, but not limited, by intramolecular cyclisation of *N*-substituted aromatic ortho-diamines [10], ring transformation of benzofurazans [11], and condensation of benzofuran-1-oxide to form quinoxaline-*N*-oxides [12]. The most common method for the preparation of quinoxaline derivatives relies on the condensation of an aryl 1,2-diamine with a 1,2-dicarbonyl compound, of which this type of reaction has limitations due to the use of pre-defined starting materials [13,14,15,16], which limit the number of substituents that can be added.

A more convenient approach for the synthesis of quinoxaline derivatives is the substitution of hydrogen at the 2- or 3-position of quinoxaline by *C*-nucleophiles. Substitution of hydrogen by *C*-nucleophiles on electron deficient arenes has been reported extensively [17,18,19]. The reaction of quinoxaline derivatives with aryl and alkyl nucleophiles has been investigated [20,21,22,23,24,25,26]. *C*-nucleophiles have been shown to add to 6-aminoquinoxaline [9,20] affording alkyl- and aryl-substituted quinoxalines in low to high yields. Although efficient, these reactions require the use of an oxidising agent to afford aromatic substitution products. Azev et al. [21], reported formation of resorcinol derivative, 2-(2,4-dihydroxyphenyl)quinoxaline after reacting quinoxaline and resorcinol in refluxing ethanol (EtOH) in the presence of an acid. The reaction of quinoxaline with this nucleophile continues to oxidative rearomatization of the compound without the need of having an oxidizing agent, but requires the use of concentrated HCl and high temperature. The use of nucleophilic substitution has been extended to other functional groups like aromatic amines and heteroaryls, but examples of quinoxaline derivatives containing these groups are limited. The reaction of 2-chloro-3-methylquinoxaline with aromatic amines as nucleophilic reagents in the presence of anhydrous potassium carbonate and potassium iodide, forming the corresponding 2-arylamino-3-methylquinoxalines, has been described by Badr et al. [22]. This method only describes 2-arylaminoquinoxaline derivatives containing a methyl-substituent at the 3-position. A more recent strategy by Zhou entails direct heteroarylation of *N*-heteroarenes by heteroaryl Grignard reagents. Although this method gives high yields and is efficient, it focuses solely on heterobiaryl preparation for double functionalization [23,25,26]. Phenylacetylide nucleophile was also reported to add to quinoxaline via nucleophilic substitution, affording mono-substituted and di-substituted quinoxaline [24]. The influence of substituents at the 2-position on nucleophilic substitution by *C*-nucleophiles has not been fully investigated. Herein, we wish to report nucleophilic substitution reactions on mono-substituted quinoxalines, bearing an aryl, alkyl, alkynyl, and amino substituents at the 2-position.

By starting with quinoxaline bearing a benzenesulfonyloxy leaving group, which we previously reported to be an efficient leaving group in coupling of pteridines [27,28], we envisaged that we can prepare mono-substituted quinoxaline derivatives, employing the Negishi [29], Sonogashira [30], and Buchwald coupling conditions [31].

## 2. Results

**Scheme 1:**
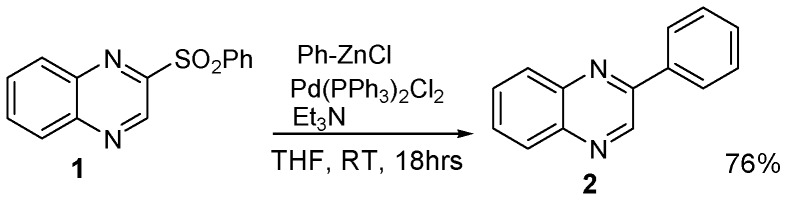
Negishi Coupling.

Our investigation began with the preparation of 2-benzensulfonyloxyquinoxaline **1** which was prepared from our previously reported method [27]. 2-Phenylquinoxaline **2** was prepared via Negishi coupling of **1** and 1.14 M solution of phenyl-ZnCl, in 75% yield (Scheme 1). Treating 2 with 0.5 equivalents of *n*-BuLi gave 3-butyl-2-phenylquinoxaline **2a** in 12% yield. The oxidative elimination of H_2_ occurs in atmospheric oxygen, to exclusively provide the aromatic product. Increasing the amount *n*-BuLi to 1.5 equivalents gave **2a** in 66% yield, which is a significant improvement. This method was extended to other; aryl-, heteroaryl-, alkynyl-, and alkyl-nucleophiles on compound **2**, of which the compounds **2a**–**2g** were synthesised (Table 1).

Alkyl nucleophiles afforded 2,3-disubstituted quinoxalines in higher yields compared to the aryl and alkynyl nucleophiles (entries 1, 2 vs. entries 3–7). The high electron density found on the aryl and alkynyl nucleophiles could be responsible for the low to moderate yields observed. 

We also envisaged that the phenyl group attached at the 2-position could be affecting the yields, presumably due to steric hindrance. We then synthesised 2-butylquinoxaline **3**, which contain a less bulky substituent, by Negishi coupling of **1a** and butyl-ZnCl. Reacting compound **3** with 1.5 eq of *n*-BuLi, gave 2,3-dibutylquinoxaline **3a** in 76% yield. When the number of equivalents of *n*-BuLi was increased to 3.0 eq, the target compound **3a** was isolated in 97% yield. We were also able to synthesize 2-butyl-3-(furan-2-yl)quinoxaline **3b** in 91% yield, 3-butyl-2-phenylquinoxaline **2a** in 95% yield and 3-butyl-2-(2-phenylethynyl)quinoxaline **3c** in 65% yield. The use of 2-butylquinoxaline **3** for the synthesis of **3a**–**c** and **2a** (Table 2) further supports the assumption that the low yields are due to the phenyl-group. The use of a less bulky substituent, in this case, makes nucleophilic substitution occur readily, affording products in good yields.

**Scheme 2:**
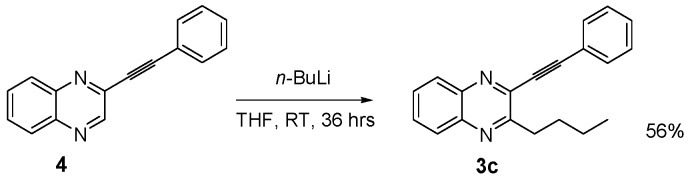
Nucleophilic substitution on 2-(2-phenylethynyl)quinoxaline.

The successful synthesis of compounds **2a**–**2g**, and **3a**–**c**, encouraged us to further investigate the possibilities of nucleophilic addition on compounds with a different functional group on the 2-position of the quinoxaline moiety. We wanted to investigate the possibilities for nucleophilic substitution if the quinoxaline moiety at the 2-position had a Csp^2^-Csp bond. We thus employed Sonogashira cross-coupling conditions to synthesise 2-(2-phenylethynyl)quinoxaline **4**. Treating **4** with 3.0 equivalents of *n*-BuLi gave 3-butyl-2-(2-phenylethynyl)quinoxaline **3c** in 20% yield. Increasing the reaction time from 18 h to 36 h improved the percentage yield to 56% (Scheme 2). 

Attempts to extend the scope to other *C*-nucleophiles employed on quinoxaline **2**, were unsuccessful as only the starting material was recovered. We postulate that nucleophilic substitution on 2-(2-phenylethynyl)quinoxaline **4** was difficult due to the electron donating nature of the phenylalkynyl attached at the 2-position of the quinoxaline moiety. This is possibly due to the high electron density caused by highly delocalized pi-electrons from the phenyl ring and the acetynyl moiety, which could make the –N=C–H site less electrophilic. This suggests that only very strong nucleophiles, such as *n*-BuLi, can work efficiently. We then proceeded to investigate the possibility of nucleophilic attack if the bonding atom on the 2-position of the quinoxaline was C–N. We used Buchwald-Hartwig cross-coupling reaction to synthesize quinoxaline derivatives containing an arylamine.

The coupling reaction between 2-benzenesulfonyloxyquinoxaline **1** and aniline, under Buchwald-Hartwig conditions, formed *N*-phenylquinoxalin-2-amine **5**. When we extended nucleophilic substitution on the compounds synthesized by Buchwald-Hartwig coupling, we found that the results were similar to those on the Sonogashira coupled product **4**. When *N*-phenylquinoxalin-2-amine **5** was reacted with *n*-BuLi (3.0 eq) and left to stir for 18 h, the target compound 3-butyl-*N*-phenylquinoxalin-2-amine **5a** was isolated in 15% yield and there was also recovery of the starting material **5**. When the reaction time was increased to 36 h, compound **5a** was isolated in 32% yield (Table 3), and there was still recovery of the starting material. Reacting **5** with a less bulky nucleophile, Me-MgCl, gave 3-methyl-*N*-phenylquinoxalin-2-amine **5b** in 42% yield. The low yields could be due to the competition between deprotonation of the free N–H and nucleophilic substitution at the –N=C–H site. The N–H group on compound **5**, was methylated to give *N*-methyl-*N*-phenylquinoxalin-2-amine **6**.

Reacting quinoxaline **6** with *n*-BuLi gave 3-butyl-*N*-methyl-*N*-phenylquinoxalin-2-amine **6a** in 92% yield and no starting material was recovered. With the protecting group (methyl-) on the nitrogen, the *n*-BuLi was able to fully react with the starting material **6** with no other competing reactions (Table 3). Attempts to use other nucleophiles on the mono-substituted Buchwald-Hartwig coupled products were not successful. Similarly to the Sonogashira coupled product **4**, only strong alkyl nucleophiles can readily react at the 3-position of the quinoxaline moiety. This could be due to steric hindrance accompanied by electron density on nitrogen atom, making the –N=C–H site less reactive towards nucleophiles.

In conclusion, nucleophilic substitution on 2-phenylquinoxaline **2** and 2-butylquinoxaline **3**, proceeded smoothly to give 2,3-disubstituted quinoxalines. Di-substituted quinoxalines bearing aryl-alky, aryl-aryl, aryl-heteroaryl, and aryl-alkynyl substituents were prepared in moderate to good yields. Nucleophilic substitutions on 2-acetylene and 2-amine quinoxaline, were only possible with strong alkyl nucleophiles (*n*-BuLi and Me-MgCl). This could be due to steric and electronic properties associated with these substituents. Molecular modelling studies are being conducted to understand the influence of the substituent attached at the 2-position, and the nature of the nucleophile. 

## 3. Experimental Section

### 3.1. General Information

Reagents were purchased from Sigma-Aldrich (Johannesburg, South Africa) and were used without further purification. Melting points were obtained using Lasec/SA-melting point apparatus from Lasec Company, SA (Johannesburg, South Africa). ^1^H- and ^13^C-NMR spectra were recorded at 400 MHz and 100 MHz, respectively, on Bruker Avance-400 spectrometer (Johannesburg, South Africa). Spectra were recorded in deuterated chloroform (CDCl_3_) unless otherwise specified. Chemical shifts are reported in parts per million (ppm) relative to tetramethylsilane as an internal standard at zero ppm. The ^1^H-NMR chemical shifts are reported: value (number of hydrogens, description of signal, assignment) and the ^13^C-NMR chemical shifts are reported: value (assignment). Abbreviations used: s = singlet, d = doublet, dd = doublet of doublets, t = triplet, and m = multiplet.

### 3.2. Synthetic Procedure

#### 3.2.1. 2-Benzensulfonyloxyquinoxaline (**1**)

In a round bottom flask, quinoxalinone (5 g, 34 mmol), DMAP (0.416 g, 3.4 mmol) and benzenesulfonyl chloride (8.72 mL, 68 mmol) were dissolved in DCM (100 mL), cooled to 0 °C and stirred for 5 min. Et_3_N (12 mL, 88 mmol) was added drop-wise over 5 min, the solution allowed to stir at room temperature for 1 h, and the reaction quenched with aqueous NaHCO_3_ (80 mL). The two layers were separated and the aqueous layer washed twice with DCM (2 × 60 mL). The combined organic layers were dried over MgSO_4_, filtered, concentrated, and dissolved in EtOAc (15 mL). The filtrate was concentrated to give 2-benzenesulfonyloxyquinoxaline as a brown solid (8.23 g, 85%); m.p. 90–91 °C; δ_H_ (400 MHz, CDCl_3_) 7.58–7.63 (2H, m), 7.69–7.77 (3H, m), 7.87–7.90 (1H, m), 8.09–8.17 (3H, m) and 8.67 ppm (1H, s); δ_C_ (100 MHz, CDCl_3_) 128.51, 128.80, 129.01, 129.22, 129.51, 131.20, 134.74, 136.41, 139.10, 139.71, 141.32 and 150.90; HRMS (ES): MH^+^ calcd for [C_14_H_10_N_2_SO_3_]^+^: 286.04212, found: 286.0421.

#### 3.2.2. 2-Phenylquinoxaline (**2**)

ZnCl_2_ (0.95 g, 6.99 mmol, 2.0 eq, taken directly from the oven) was added to an oven dried 2-neck flask with a stirrer bar, which was further dried under vacuum for 15 min. Dry THF (10 mL) was added to the flask under dry nitrogen. The solution was left to stir until all dried ZnCl_2_ was dissolved. Phenyl-MgBr (6.3 mL, 6.29 mmol, 1 M) was added to the solution and left to stir for 1 h. To a solution of Phenyl-ZnCl (6.29 mmol), 2-benzenesulfonyloxyquinoxaline **1** (1 g, 3.49 mmol) and PdCl_2_(PPh_3_)_2_ (122.6 mg, 0.18 mmol, 5 mol %) were added and set to reflux while stirring under nitrogen for 20 h. The solution was allowed to cool to room temperature, diluted with EtOAc (25 mL), quenched with sat. NaHCO_3_ (15 mL). The two layers were separated and the aqueous layer diluted with EtOAc (15 mL). The combined organic layers were dried over Na_2_SO_4_, filtered and concentrated, and purified on silica gel eluting with 20% EtOAc/Hexane to give 2-phenylquinoxaline as a yellow solid (0.537 g, 75%); m.p. 73–74 °C (Lit. 74–75 °C); δ_H_ (400 MHz, CDCl_3_) 7.46–7.52 (3H, m), 7.69–7.77 (2H, m), 8.12–8.15 (4H, m) and 9.27 (1H, s); δ_C_ (100 MHz, CDCl_3_) 127.63, 128.54, 129.32, 129.51, 129.65, 130.58, 138.87, 132.16, 136.24, 140.41, 142.51 and 152.00; *m/z* (ES-API, +ve) 207.1 (M + 1100). Physical and spectroscopic data agree with those reported in literature [32].

##### General Methods for Nucleophilic Substitution on 2-Phenylquinoxaline

**Method 1**: A solution of *n*-BuLi or Mg-aryl/alkyl was treated with 2-phenylquinoxaline **2** (100 mg, 0.485 mmol), and the solution allowed to stir at room temperature for 18 h. The final solution was diluted with EtOAc (10 mL), quenched with sat. NaHCO_3_ (10 mL). The organic layers were combined and dried over Na_2_SO_4_, filtered, concentrated, and purified by flash column chromatography on silica gel.

**Method 2**: A solution of either an alkyne or hetero-aromatic substrate (0.97 mmol) in THF (5 mL) was lithiated with *n*-BuLi (0.4 mL, 0.97 mmol, 2 eq, 2.5 M) and left to stir for 15 min under a nitrogen atmosphere. 2-Phenylquinoxaline **2** (100 mg, 0.485 mmol) was added to the flask, and the solution allowed to stir at room temperature for 18 h. The final solution was diluted with EtOAc (10 mL), quenched with sat. NaHCO_3_ (10 mL). The organic layers were combined and dried over Na_2_SO_4_, filtered, concentrated, and purified by flash column chromatography on silica gel.

#### 3.2.3. 3-Butyl-2-phenylquinoxaline (**2a**)

Following general method 1, 2-phenylquinoxaline 2 (100 mg, 0.485 mmol) was treated with *n*-BuLi (0.3 mL, 0.728 mmol, 1.5 eq, 2.5 M) at −78 °C under nitrogen atmosphere. The solution was left to stir for 18 h at room temperature, followed by aqueous work-up and purification by flash column chromatography on silica gel, eluting with 5% EtOAc/hexane to give 3-butyl-2-phenylquinoxaline as tan oil (84 mg, 66%); δ_H_ (400 MHz, CDCl_3_) 0.75–0.79 (3H, t, *J* = 7.4 Hz); 1.18–1.27 (2H, m), 1.62–1.65 (2H, m), 2.96–2.99 (2H, t, *J* = 7.8 Hz), 7.44–7.54 (5H, m), 7.65–7.68 (2H, m) and 8.01–8.06 (2H, m); δ_C_ (100 MHz, CDCl_3_) 13.83, 22.62, 29.72, 31.25, 35.76, 128.45, 128.57, 128.86, 129.22, 129.27, 129.73, 139.11, 140.72, 141.32, 146.38, 155.12 and 156.35; *m*/*z* (ES-API, +ve) 263.1 (M + 1100). Physical and spectroscopic data agree with those reported in literature [14].

#### 3.2.4. 3-Isopropyl-2-phenylquinoxaline (**2b**)

Following general method 1, 2-phenylquinoxaline **2** (100 mg, 0.485 mmol) was treated with ^*i*^PrMgCl (0.8 mL, 0.728 mmol, 1.5 eq, 0.9 M) at 0 °C under nitrogen atmosphere. The solution was left to stir for 18 h, followed by aqueous work-up and purification by flash column chromatography on silica gel, eluting with 5% EtOAc/hexane to give 2-isopropyl-3-phenylquinoxaline as yellowish solid (65.28 mg, 54%); m.p. 97–98 °C (Lit. 98–99 °C); δ_H_ (400 MHz, CDCl_3_) 1.17–1.25 (6H, d, *J* = 6.8 Hz), 3.39–3.46 (1H, m), 7.41–7.51 (5H, m), 7.61–7.68 (2H, m) and 8.03–8.05 (2H, m); δ_C_ (100 MHz, CDCl_3_) 21.80, 30.99, 127.50, 127.66, 127.71, 127.79, 128.07, 128.18, 128.51, 138.12, 139.42, 140.66, 153.53 and 159.74; *m/z* (ES-API, +ve) 249.1 (M + 1100). Physical and spectroscopic data agree with those reported in literature [33].

#### 3.2.5. 2,3-Diphenylquinoxaline (**2c**)

Following general method 1, 2-phenylquinoxaline **2** (100 mg, 0.485 mmol) was treated with Phenyl-MgBr (0.64 mL, 0.728 mmol, 1.5 eq, 1.14 M) at 0 °C under nitrogen atmosphere. The solution was left to stir for 18 h, followed by aqueous work-up and purification by flash column chromatography on silica gel, eluting with 5% EtOAc/hexane to give 2,3-diphenylquinoxaline as orange solid (29.8 mg, 22%); m.p. 126–128 °C (Lit. 127–129 °C); δ_H_ (400 MHz, CDCl_3_) 7.27–7.29 (6H, m), 7.44–7.52 (4H, d, *J* = 7.4 Hz), 7.62–7.73 (2H, dd, *J* = 8.7 and 2.3 Hz) and 8.05–8.13 (2H, dd, *J* = 8.7 and 2.3 Hz); δ_C_ (100 MHz, CDCl_3_) 126.88, 127.47, 127.91, 128.05, 129.12, 137.73, 140.01 and 152.38; *m/z* (ES-API, +ve) 283.1 (M + 1100). Physical and spectroscopic data agree with those reported in literature [34].

#### 3.2.6. 2-Phenyl-3-(2-phenylethynyl)quinoxaline (**2d**)

Following general method 2, the resulting solution from phenylacetylene (0.1 mL, 0.97 mmol, 2.0 eq) and *n*-BuLi, was treated with **2**. After normal work-up and purification, eluting with 10% EtOAc/hexane gave 2-phenyl-3-(2-phenylethynyl)quinoxaline as light brown solid (69.2 mg, 47%); m.p. 110–112 °C (Lit. 109–111 °C); δ_H_ (400 MHz, CDCl_3_) 7.33–7.39 (3H, m), 7.48–7.50 (2H, d, *J* = 6.8 Hz), 7.56–7.57 (3H, m), 7.77–7.79 (2H, m) and 8.09–8.15 (4H, m); δ_C_ (100 MHz, CDCl_3_) 87.31, 94.06, 120.66, 127.15, 127.44, 127.74, 128.29, 128.60, 128.67, 128.71, 129.31, 129.69, 131.11, 136.60, 137.08, 139.70, 139.99 and 154.10; *m/z* (ES-API, +ve) 307.1 (M + 1100). Physical and spectroscopic data agree with those reported in literature [35].

#### 3.2.7. 3-(Oct-1-ynyl)-2-phenylquinoxaline (**2e**)

Following general method 2, the resulting solution from 1-octyne (0.14 mL, 0.97 mmol, 2.0 eq) and *n*-BuLi was treated with **2**. After normal work-up and purification, eluting with 10% EtOAc/hexane gave 2-(oct-1-ynyl)-3-phenylquinoxaline as orange oil (20.5 mg, 14%); δ_H_ (400 MHz, CDCl_3_) 0.86–0.89 (3H, t, *J* = 6.8 Hz), 1.25–1.32 (4H, m), 1.52–1.60 (4H, m), 2.42–2.46 (2H, t, *J* = 7.1 Hz), 7.50–7.52 (3H, m), 7.74–7.76 (2H, m) and 8.02–8.03 (4H, m); δ_C_ (100 MHz, CDCl_3_) 14.10, 19.76, 22.53, 27.81, 28.64, 29.72, 31.35, 97.92, 128.08, 128.64, 129.23, 129.53, 129.58, 130.13, 130.36, 137.75, 138.50, 140.60,140.93 and 154.92. HRMS (ES): MH^+^ calcd for [C_22_H_22_N_2_]^+^: 315.1783, found: 315.1861.

#### 3.2.8. 2-Phenyl-3-(thiophen-2-yl)quinoxaline (**2f**)

Following general method 2, the resulting solution from thiophene (0.08 mL, 0.97 mmol, 2.0 eq) and *n*-BuLi was treated with **2**. After normal work-up and purification, eluting with 10% EtOAc/hexane gave 2-phenyl-3-(thiophen-2-yl)quinoxaline as a lime solid (30.5 mg, 22%); m.p. 126–128 °C (Lit. 128 °C); δ_H_ (400 MHz, CDCl_3_) 6.75–6.76 (1H, d, *J* = 3.6 Hz), 6.86–6.88 (1H, m), 7.40–7.41 (1H, d, *J* = 4.8 Hz), 7.48–7.54 (3H, m), 7.59–7.61 (2H, m), 7.70–7.79 (2H, m) and 8.08–8.12 (2H, m); δ_C_ (100 MHz, CDCl_3_) 127.78, 128.76, 128.85, 129.07, 129.14, 129.25, 129.34, 129.77, 129.95, 130.23, 139.42, 140.45, 141.14, 142.74, 146.91 and 152.58; *m/z* (ES-API, +ve) 289.1 (M + 1100). Physical and spectroscopic data agree with those reported in literature [36].

#### 3.2.9. 2-(Furan-2-yl)-3-phenylquinoxaline (**2g**)

Following general method 2, the resulting solution from furan (0.08 mL, 0.97 mmol, 2.0 eq) and *n*-BuLi was treated with **2**. After normal work-up and purification, eluting with 10% EtOAc/hexane gave 2-(furan-2-yl)-3-phenylquinoxaline as brown solid (60.6 mg, 46%); m.p. 102–103 °C (Lit. 103–104 °C); δ_H_ (400 MHz, CDCl_3_) 6.14–6.15 (1H, d, *J* = 3.5 Hz), 6.35–6.36 (1H, m), 7.50–7.59 (6H, m), 7.71–7.79 (2H, m), 8.10–8.12 (1H, dd, *J* = 8.0 and 1.1 Hz) and 8.20–8.28 (1H, dd, *J* = 8.0 and 1.1 Hz); δ_C_ (100 MHz, CDCl_3_) 111.79, 114.88, 128.70, 128.82, 129.07, 129.13, 129.25, 129.96, 130.35, 139.28, 140.35, 141.01, 143.09, 144.74, 150.88 and 152.57; *m/z* (ES-API, +ve) 273.1 (M + 1100). Physical and spectroscopic data agree with those reported in literature [37].

#### 3.2.10. 2-Butylquinoxaline (**3**)

ZnCl_2_ (475.7 mg, 3.49 mmol, 2.0 eq, taken directly from the oven) was added to a pre-oven dried round bottom flask with a stirrer bar. Dry THF (20 mL) was added to the flask and left to stir until all ZnCl_2_ was dissolved, under nitrogen. *n*-BuLi (1.4 mL, 3.49 mmol, 2.0 eq, 2.5 M) was added to the flask at −78 °C, left to stir for 1 h at −78 °C. To the solution, 2-benzenesulfonyloxyquinoxaline **1** (500 mg, 1.75 mmol) and PdCl_2_(PPh_3_)_3_ (61.3 mg, 0.0874 mmol, 5 mol %) were added and set to reflux while stirring under nitrogen for 18 h. The solution was allowed to cool to room temperature, diluted with EtOAc (12.5 mL) and quenched with sat. NaHCO_3_ (2 × 15 mL). The two layers separated and the aqueous layer diluted with EtOAc (8 mL). The combined organic layers were dried over Na_2_SO_4_, concentrated, and purified on silica gel eluting with 10% EtOAc/Hexane to give 2-butylquinoxaline as an orange oil (200 mg, 61%); δ_H_ (400 MHz, CDCl_3_) 1.00 (3H, t, *J* = 7.4 Hz), 1.44–1.49 (2H, m), 1.79–1.86 (2H, m), 3.02 (2H, t, *J* = 7.8 Hz), 7.70–7.74 (2H, m), 8.03–8.08 (2H, m) and 8.75 (1H, s); δ_C_ (100 MHz, CDCl_3_) 14.13, 22.62, 31.61, 36.37, 128.94, 129.16, 129.29, 139.99, 141.26, 142.20, 145.84 and 157.73; *m/z* (ES-API, +ve) 188 (M + 1100). Physical and spectroscopic data agree with those reported in literature [38].

#### 3.2.11. 2,3-Dibutylquinoxaline (**3a**)

Following general method 1, 2-butylquinoxaline **3** (50 mg, 0.269 mmol) was treated with *n*-BuLi (0.32 mL, 0.807 mmol, 3.0 eq, 2.5 M) at −78 °C under nitrogen atmosphere. The solution was left to stir for 18 h at room temperature, followed by aqueous work-up and purification by flash column chromatography on silica gel, eluting with 10% EtOAc/hexane to give 2,3-dibutylquinoxaline as brown oil (63 mg, 97%); δ_H_ (400 MHz, CDCl_3_) 0.89–1.11 (6H, t, *J* = 7.4 Hz), 1.49–1.53 (4H, m), 2.99–3.03 (4H, t, *J* = 7.4 Hz), 7.63–7.66 (2H, m) and 7.98–8.00 (2H, m); δ_C_ (100 MHz, CDCl_3_) 14.01, 22.91, 31.05, 35.21, 128.47, 128.69, 140.99 and 156.72. Physical and spectroscopic data agree with those reported in literature [39].

#### 3.2.12. 2-Butyl-3-(furan-2-yl)quinoxaline (**3b**)

Furan (0.06 mL, 0.807 mmol, 3.0 eq) was treated with *n*-BuLi (0.32 mL, 0.807 mmol, 3.0 eq, 2.5 M) at −78 °C under nitrogen atmosphere. The resulting solution was treated with **3** and the solution was left to stir for 18 h at room temperature, followed by aqueous work-up and purification by flash column chromatography on silica gel, eluting with 10% EtOAc/hexane to give 2-butyl-3-(furan-2-yl)quinoxaline as brown oil (62 mg, 91%); δ_H_ (400 MHz, CDCl_3_) 0.97–1.01 (3H, t, *J* = 7.4 Hz), 1.48–1.54 (2H, m), 1.78–1.84 (2H, m), 3.29–3.33 (2H, t, *J* = 7.8 Hz), 6.63–6.64 (1H, m), 7.22–7.23 (1H, d, *J* = 3.5 Hz), 7.68–7.71 (3H, m) and 8.01–8.10 (2H, m); δ_C_ (100 MHz, CDCl_3_) 14.01, 22.87, 30.81, 36.69, 112.12, 113.96, 128.41, 128.93, 129.41, 129.62 and 144.49. HRMS (ES): MH^+^ calcd for [C_16_H_16_N_2_O]^+^: 253.1263, found: 253.1339.

#### 3.2.13. 3-Butyl-2-(2-phenylethynyl)quinoxaline (**3c**)

Following general method 2, the resulting solution from phenylacetylene (0.1 mL, 0.97 mmol, 2.0 eq) and *n*-BuLi, was treated with **3**. After normal work-up and purification, eluting with 10% EtOAc/hexane gave 3-butyl-2-(2-phenylethynyl)quinoxaline as orange solid (90.1 mg, 65%); m.p. 76–78 °C; δ_H_ (400 MHz, CDCl_3_) 0.93–0.96 (3H, t, *J* = 7.4 Hz), 1.44–1.50 (2H, m), 1.82–1.89 (2H, m), 3.19–3.23 (2H, t, *J* = 7.8 Hz), 7.35–7.37 (3H, m), 7.60–7.66 (4H, m) and 7.95–8.97 (2H, m); δ_C_ (100 MHz, CDCl_3_) 14.02, 22.90, 29.72, 30.93, 36.34, 86.87, 94.90, 121.74, 128.62, 128.84, 129.51, 129.70, 130.34, 132.24, 139.52, 140.73,140.85 and 158.86. HRMS (ES): MH^+^ calcd for [C_20_H_18_N_2_]^+^: 287.1470, found: 287.1545.

#### 3.2.14. 2-(2-Phenylethynyl)quinoxaline (**4**)

Under a nitrogen atmosphere, in a round bottom flask equipped with a stirrer bar, 2-benzenesulfonyloxyquinoxaline **1** (0.301 g, 1.05 mmol), PdCl_2_(PPh_3_)_2_ (38.0 mg, 0.052 mmol, 5 mol %), Et_3_N (0.2 mL, 2.1 mmol, 2 eq) and phenylacetylene (154 µL, 1.26 mmol, 1.2 eq) were dissolved in THF (10 mL). The solution was refluxed for 18 h under nitrogen, followed by aqueous work-up and purification by flash column chromatography on silica gel, eluting with 1:9 EtOAc/*n*-hexane to give 2-(2-phenylethynyl)quinoxaline as a yellowish solid (0.178 g, 61%); m.p. 65–66°C (Lit. 65–66 °C); δ_H_ (400 MHz, CDCl_3_) 7.42–7.45 (3H, m), 7.69–7.71 (2H, m), 7.78–7.82 (2H, m), 8.11–8.12 (2H, m) and 8.99 (1H, s); δ_C_ (100 MHz, CDCl_3_) 121.41, 128.58, 129.05, 129.19, 129.23, 129.27, 129.80, 130.45, 130.73, 132.40, 139.14, 139.62, 140.92, 142.22 and 147.36; *m/z* 231 (M + 1100). Physical and spectroscopic data agree with those reported in literature [40].

#### 3.2.15. *N*-Phenylquinoxalin-2-amine (**5**)

In a 50 mL 2-neck round bottom flask equipped with a stirrer bar under nitrogen, 2-benzenesulfonyloxyquinoxaline **1** (100 mg, 0.349 mmol), Pd(OAc)_2_ (4 mg, 0.0175 mmol, 5 mol %), Brett-Phos (9.4 mg, 0.0175 mmol, 5 mol %) and aniline (0.1 mL, 1.047 mmol, 3.0 eq) were dissolved in 1,4-dioxane (5 mL). Pyridine (0.08 mL, 1.047 mmol, 3.0 eq) was added to the solution, and refluxed for 18 h. The solution was diluted with EtOAc (10 mL), and quenched with sat. NaHCO_3_ (10 mL). The organic layers were combined and dried over Na_2_SO_4_, filtered then concentrated and purified by flash column chromatography on silica gel eluting with 40% EtOAc/hexane and on prep TLC with 5% MeOH/DCM to give *N*-phenylquinoxalin-2-amine as yellowish solid (40 mg, 52%); m.p. 135–137 °C (Lit. 137 °C); δ_H_ (400 MHz, CDCl_3_) 7.08 (1H, s), 7.14–7.17 (1H, m), 7.40–7.45 (2H, m), 7.48–7.52 (1H, m), 7.64–7.68 (1H, m), 7.76–7.97 (3H, m) and 8.47 (1H, s); δ_C_ (100 MHz, CDCl_3_) 119.85, 123.59, 125.65, 126.95, 128.83, 129.32, 130.37, 137.84, 138.48, 139.21, 141.19 and 149.32. *m/z* (ES-API, +ve) 222.1 (M + 1100). Physical and spectroscopic data agree with those reported in literature [41].

#### 3.2.16. 3-Butyl-*N*-phenylquinoxalin-2-amine (**5a**)

2-Quinoxalineamine **5** (50 mg, 0.226 mmol) was treated with *n*-BuLi (0.3 mL, 0.678 mmol, 3 eq, 2.5 M) at −78 °C under nitrogen atmosphere. The solution was left to stir for 36 h at room temperature, followed by aqueous work-up and purification by flash column chromatography on silica gel eluting with 10% EtOAc/hexane to give 3-butyl-*N*-phenylquinoxalin-2-amine as orange solid (22 mg, 32%); m.p. 99–102 °C; δ_H_ (400 MHz, CDCl_3_) 0.95–0.98 (3H, t, *J* = 7.4 Hz), 1.46–1.51 (2H, m), 1.81–1.89 (2H, m), 2.87–2.91 (2H, t, *J* = 7.8 Hz), 6.68 (1H, s), 7.03–7.06 (1H, m), 7.32–7.41 (3H, m), 7.48–7.52 (1H, m) and 7.72–7.83 (4H, m); δ_C_ (100 MHz, CDCl_3_) 14.03, 22.78, 28.91, 33.94, 119.85, 123.17, 125.45, 126.68, 128.17, 129.07, 137.51, 138.25, 139.44, 140.25, 147.58 and 148.01. HRMS (ES): MH^+^ calcd for [C_18_H_19_N_3_]^+^: 278.1579, found: 278.1659.

#### 3.2.17. 3-Methyl-*N*-phenylquinoxalin-2-amine (**5b**)

2-Quinoxalineamine **5** (50 mg, 0.226 mmol) was treated with Me-MgCl (0.3 mL, 3 eq, 3.0 M) at 0 °C under nitrogen atmosphere. The solution was left to stir for 36 h at room temperature, followed by aqueous work-up and purification by flash column chromatography on silica gel eluting with 10% EtOAc/hexane to give 3-methyl-*N*-phenylquinoxalin-2-amine as orange oil (22 mg, 42%); δ_H_ (400 MHz, CDCl_3_) 2.43 (3H, s), 6.61 (1H, s), 7.39–7.41 (2H, m), 7.45–7.48 (2H, m), 7.53–7.57 (3H, m), 7.94–7.97 (1H, m) and 8.09–8.12 (1H, m); δ_C_ (100 MHz, CDCl_3_) 15.32, 116.36, 119.22, 124.82, 125.44, 128.34, 128.82, 129.63, 136.44, 140.28, 143.62, 145.99 and 160.32. Physical and spectroscopic data agree with those reported in literature [23].

#### 3.2.18. *N*-Methyl-*N*-phenylquinoxalin-2-amine (**6**)

2-Quinoxalineamine **5** (200 mg, 0.904 mmol) was dissolved in THF (20 mL) and was treated with NaH (43 mg, 1.81 mmol, 2.0 eq) at 0 °C and left to stir for 1 h at room temperature. Iodomethane (0.2 mL, 0.712 mmol, 3.0 eq) was added to the solution under nitrogen. The solution was left to stir for 5 h at room temperature. The solution was quenched with sat. NH_4_Cl and purified by flash column chromatography on silica gel eluting with 5% MeOH/DCM to give *N*-methyl-*N*-phenylquinoxalin-2-amine as orange oil (165 mg, 78%); δ_H_ (400 MHz, CDCl_3_) 3.60 (3H, s), 7.32–7.34 (3H, m), 7.38–7.43 (1H, m), 7.45–7.49 (2H, m), 7.58–7.62 (1H, m), 7.76–7.88 (2H, m) and 8.27 (1H, s); δ_C_ (100 MHz, CDCl_3_) 36.68, 124.83, 126.42, 126.57, 126.82, 128.76, 128.76, 130.02, 130.23, 136.92, 141.67, 144.96 and 152.20. HRMS (ES): MH^+^ calcd for [C_15_H_13_N_3_]^+^: 236.1109, found: 236.1191.

#### 3.2.19. 3-Butyl-*N*-methyl-*N*-phenylquinoxalin-2-amine (**7a**)

*N*-Methyl-*N*-phenylquinoxalin-2-amine **6** (50 mg, 0.213 mmol) was treated with *n*-BuLi (0.26 mL, 0.639 mmol, 3 eq, 2.5 M) at −78 °C under nitrogen atmosphere. The solution was left to stir for 36 h at room temperature, followed by aqueous work-up and purification by flash column chromatography on silica gel eluting with 10% EtOAc/hexane to give 3-butyl-*N*-methyl-*N*-phenylquinoxalin-2-amine as yellow oil (52.2 mg, 62%); δ_H_ (400 MHz, CDCl_3_) 0.75–0.79 (3H, t, *J* = 7.4 Hz), 1.09–1.19 (2H, m), 1.52–1.58 (2H, m), 2.36–2.40 (2H, t, *J* = 7.8 Hz), 3.55 (1H, s), 6.97–6.99 (2H, m), 7.10–7.14 (1H, m), 7.28–7.34 (2H, m), 7.51–7.56 (1H, m), 7.60–7.63 (1H, m), 7.86–7.89 (1H, d, *J* = 7.6 Hz) and 7.92–7.94 (1H, d, *J* = 7.4 Hz); δ_C_ (100 MHz, CDCl_3_) 13.84, 22.56, 29.78, 35.44, 41.73, 122.96, 124.33, 126.66, 127.05, 127.97, 128.81, 129.62, 138.83, 140.29, 148.92, 153.34 and 154.28. HRMS (ES): MH^+^ calcd for [C_19_H_21_N_3_]^+^: 292.1735, found: 292.1817.

## Figures and Tables

**Table 1:**
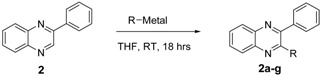
Nucleophilic substitution on 2-phenylquinoxaline.

Entry	Product	R-Metal	R	% Yield
1	**2a**	*n*-BuLi	-Butyl	66
2	**2b**	Isopropyl-MgCl	-Isopropyl	54
3	**2c**	Phenyl-MgBr	-Phenyl	22
4	**2d**	2-Phenylethynyl-Li	-2-Phenylethynyl	47
5	**2e**	Oct-1-ynyl-Li	-Oct-1-ynyl	14
6	**2f**	Thiopen-2-yl-Li	-Thiophen-2-yl	22
7	**2g**	Furan-2-yl-Li	-furan-2-yl	46

A solution of Li- or Mg-aryl/alkyl was treated with 2-phenylquinoxaline **2** (100 mg, 0.485 mmol), and the solution allowed to stir at room temperature for 18 h. The final solution was diluted with EtOAc (10 mL), quenched with sat. NaHCO_3_ (10 mL). The organic layers were combined and dried over Na_2_SO_4_, filtered, concentrated, and purified by flash column chromatography on silica gel.

**Table 2:**
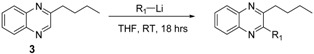
Nucleophilic substitution on 2-butylquinoxaline.

Entry	Product	R^1^	% Yield
1	**3a**	-Butyl	97
2	**3b**	-Furan-2-yl	91
3	**2a**	-Phenyl	95
4	**3c**	-Phenylacetylene	65

**Table 3:**
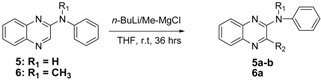
Nucleophilic substitution on *N*-benzylquinoxalin-2-amine.

Entry	Product	R^1^	R^2^	% Yield
1	**5a**	-H	-Butyl	32
2	**5b**	-H	-Methyl	42
3	**6a**	-CH_3_	-Butyl	62
